# A case of pediatric Perthes’ disease with unexplained hyperlactatemia at the time of initial surgery and anesthetic management with remimazolam for the subsequent surgery

**DOI:** 10.1186/s40981-024-00715-2

**Published:** 2024-05-24

**Authors:** Ko Ishikawa, Tadanao Hiroki, Sachiko Ito, Chizu Aso, Shigeru Saito

**Affiliations:** 1 Department of Anesthesiology, Isesaki Municipal Hospital, 12-1, Tsunatorihon-Machi, Isesaki, Gunma 372-0817 Japan; 2 Department of Anesthesiology, Japanese Red Cross Maebashi Hospital, Maebashi, Japan; 3 Department of Anesthesiology, Gunma University Hospital, Maebashi, Japan

**Keywords:** Hyperlactatemia, Lactic acidosis, Lactate, Remimazolam, Pediatric

## Abstract

**Background:**

The causes of perioperative hyperlactatemia vary, but they are generally associated with hypoperfusion. Here, we report the case of a pediatric patient who developed unexplained hyperlactatemia during anesthesia with propofol and sevoflurane, which recurred during a second surgery under anesthesia with remimazolam.

Case presentation.

An 8-year-old boy with Perthes disease and no remarkable past or family history was scheduled for an osteotomy. Anesthesia was induced with propofol and rocuronium and then maintained with sevoflurane and remifentanil. The patient developed lactic acidosis without hemodynamic instability during anesthesia, with a normal lactate/pyruvate ratio after surgery, suggesting a lack of hypoperfusion. We used remimazolam instead of propofol during the second surgery 6 months later, considering the possibility of drug-induced lactic acidosis, including malignant hyperthermia and propofol infusion syndrome, where the unexplained hyperlactatemia recurred.

**Conclusions:**

Distinguishing the causes of hyperlactatemia, particularly in the absence of other symptoms, is challenging. The lactate/pyruvate ratio during episodes of hyperlactatemia can provide insights into the underlying pathology.

## Background

Lactic acidosis is defined as hyperlactatemia in the presence of acidosis (arterial blood pH < 7.35) [1]. Although tissue hypoperfusion is the predominant cause of perioperative hyperlactatemia, other conditions, such as drug and metabolic abnormalities, may also be responsible [2]. Propofol infusion syndrome (PRIS) and malignant hyperthermia cause hyperlactatemia, which can be fatal [3–5]. The management of perioperative hyperlactatemia should be tailored to the specific cause. This case report details hyperlactatemia in a pediatric patient who underwent anesthesia with propofol and sevoflurane during primary surgery and with remimazolam for subsequent surgery. Written informed consent was obtained from the patient’s legal guardian for the publication of this case report.

## Case presentation

An 8-year-old boy (height, 122 cm; body weight 26.9 kg; ASA physical status class I) with Perthes’ disease was scheduled for osteotomy. He had no significant medical history other than amblyopia. He had no family history of metabolic diseases and none of his family members had received general anesthesia. His blood pressure in the ward was approximately 100/60 mmHg.

Anesthesia was induced using a propofol bolus of 2 mg/kg, followed by rocuronium 0.8 mg/kg and fentanyl 0.2 μg/kg. After intubation, a radial artery catheter was placed to monitor blood pressure and hemoglobin (Hb). The patient was placed in the right-lateral recumbent position. Anesthesia was maintained with 40% oxygen and 2% sevoflurane in combination with the continuous infusion of 0.2–0.4 μg/kg/min of remifentanil. The intravenous fluids administered included Ringer’s solution with acetate, bicarbonate Ringer’s solution, and a colloidal solution; lactated Ringer’s solution was not used. Blood loss was recorded as 150 mL, and 200 mL of autologous blood was used intraoperatively. Blood gas parameters including pH, partial pressure of carbon dioxide (pCO_2_), bicarbonate (HCO_3_), Hb, and lactate levels were closely monitored. Throughout anesthesia, there was a progressive increase in lactate levels, indicative of high anion gap metabolic acidosis (Table 1). The duration of surgery was 5.5 h, and the duration of anesthesia was 8 h. No intraoperative tourniquet was used, and the systolic blood pressure remained at 80–100 mmHg. There was no increase in partial pressure of exhaled carbon dioxide and no muscle rigidity, and the patient’s temperature remained around 37.5 °C. The lactate dehydrogenase level measured intraoperatively was 116 U/L. The intraoperative fluid balance was + 2050 mL, and the urine output was maintained at 1.8 mL/kg/h (400 mL).

**Table 1:** Values of parameters during the first surgery

	After induction	2 h	3 h	5 h	6.5 h	8 h (PACU)
Lactate (mM/L)	1.7	4.9	5.9	6.3	5.7	7.5
BE (mM/L)	− 2.5	− 5.8	− 6.8	− 9.6	− 8	− 10.7
HCO_3_ (mM/L)	21.4	18.8	19.1	17.6	18.7	16.6
pH	7.41	7.34	7.34	7.26	7.23	7.24
pCO_2_ (mmHg)	34.9	35.5	35.7	39.4	39.3	38.3
Hb (g/dL)	11.3	10.8	9.1	8.9	9.6	10.6
Glu (mg/dL)	109	163	141	164	150	181
Cl (mM/L)	111	112	112	114	114	113
K (mM/L)	3.8	3.4	3.6	3.9	4.5	3.6

*Abbreviations: PACU* post-anesthesia care unit, *pCO*_*2*_ partial pressure of carbon dioxide, *Hb* hemoglobin, *Cl* chlorine, *K* potassium, *BE* base excess, *Glu* glucose

The postoperative arousal was uneventful, and the patient was extubated without complications. His laboratory data showed lactate levels within reference limits, with pH 7.4 and lactate 0.94 mmol/L the day after the surgery.

The patient was referred to the pediatric department for a thorough examination of his metabolic disease. Blood samples were collected on postoperative day 49. Laboratory analyses included fasting blood glucose, lactate/pyruvate (L/P) ratio, ketone and amino acid fractions, urinary lactate, and vitamin B1 levels. None of the differences were significant. Laboratory data suggested that the patient was unlikely to have congenital metabolic abnormalities and that it could be drug-induced or as a result of surgical invasion. We suggested muscle biopsy and genetic testing for a close examination of malignant hyperthermia and mitochondrial disease, which remained differential diagnoses; however, his parents did not wish to do so.

The patient was scheduled for nail extraction 6 months after the initial surgery. Hyperlactatemia due to hypoperfusion could not be completely ruled out; however, because it could be drug-related, an anesthesia plan was discussed in our department.

Hyperlactatemia, an initial symptom of malignant hyperthermia and PRIS, is rare, but can be fatal. Therefore, we decided not to use propofol or inhalational anesthesia. Midazolam could be a choice, but its long half-life can cause delayed emergence and respiratory depression [6]. To avoid its disadvantages and avoid the use of propofol and inhaled anesthesia, induction and maintenance of anesthesia with remimazolam were planned.

The appropriate dosage of remimazolam in pediatric patients is yet to be established, and dosages differ in each report [7, 8]. Based on these reports, we determined the dosage based on the per-body weight dosage for adults indicated in the package insert.

Remimazolam 12 mg/kg/h until sleep onset, remifentanil, and rocuronium were administered to induce general anesthesia. After intubation, the patient was placed in the right-lateral recumbent position. Anesthesia was maintained using remimazolam 1 mg/kg/h and remifentanil 0.2–0.5 μg/kg/min. Intraoperative infusions included Ringer’s acetate and bicarbonate Ringer’s solutions; no lactate Ringer’s solution was used. The duration of surgery was 82 min and the duration of anesthesia was 153 min. The intraoperative fluid balance was + 740 mL, and the urine output was 4.22 mL/kg/h (230 mL). Although the operative time was shorter than that of the initial surgery, increased lactate levels were observed (Table 2). The day after surgery, the patient’s lactate level was within the reference limits, and he was discharged from the hospital without any postoperative problems.

**Table 2:** Values of parameters during the second surgery

	After induction	1.5 h	3 h (PACU)
Lactate (mM/L)	1.2	3.2	3.8
BE (mM/L)	− 2.5	− 4.4	-5.3
HCO3 (mM/L)	21.5	20.7	21.5
pH	7.40	7.32	7.27
pCO2 (mmHg)	35.1	41.5	46
Hb (g/dL)	12	12.1	12.4
Glu (mg/dL)	111	137	169
Cl (mM/L)	113	114	109
K (mM/L)	3.7	3.2	2.8

*Abbreviations: PACU* post-anesthesia care unit, *pCO*_*2*_ partial pressure of carbon dioxide, *Hb* hemoglobin, *C1* chlorine, *K* potassium, *BE* base excess, *Glu* glucose

## Discussion

An 8-year-old boy with no significant medical history experienced lactic acidosis during anesthesia induced by propofol and sevoflurane. A subsequent comprehensive postoperative evaluation revealed no evidence of metabolic disease. Considering the potential for drug-induced lactic acidosis, remimazolam was used for anesthesia in subsequent surgeries to mitigate the effects of propofol and sevoflurane. Despite the short duration of the second surgery, lactic acidosis recurred; its etiology remains unclear. Tissue hypoxia is the most common cause of lactic acidosis during the perioperative period [1]; however, it is unlikely in this case because of stable hemodynamic conditions and the lack of an increased L/P ratio, an indicator of anaerobic/aerobic metabolism balance [13]. Tissue hypoxia secondary to hypoperfusion only increases the lactate level and not the pyruvate level, resulting in an increased L/P ratio [13]. However, it remains possible that the L/P ratio may be high at some points when it is not measured.

Among other causes of hyperlactatemia, such as drugs, toxins, and metabolic diseases [1], we first considered the possibility of drug-induced hyperlactatemia, including early representation of PRIS and malignant hyperthermia. According to a systematic review of the literature from 1950 to 2017, propofol was the most commonly used anesthetic in 286 adult patients with elevated lactate levels [9]. It also induces PRIS even after short-term infusion without hemodynamic instability [10]. The patient presented with hyperlactatemia during the second surgery without propofol, suggesting that propofol was not responsible for the hyperlactatemia. The likelihood of metabolic disease was not high based on the non-significant findings in fasting blood glucose, ketone, amino acid, urinary lactate, and vitamin B1 levels. Hyperlactatemia is also observed in patients with tumors [11, 12]; however, it was unlikely in our case, based on the unremarkable past history and thorough examination by the pediatric department. The hyperlactatemia may have resulted from an increase in glucose metabolism owing to elevated catecholamine levels in response to surgical stimuli [1]. However, during the second surgery, the patient also had hyperlactatemia despite the relatively minimally invasive and short surgery period, suggesting that there were some predisposing factors in addition to the above mechanisms.

A normal L/P ratio in the presence of hyperlactatemia suggests a parallel increase in pyruvate and lactate levels, which may indicate deficiencies in pyruvate dehydrogenase or gluconeogenesis [14, 15]. For postoperative examination, measuring the L/P ratio in hyperlactatemic blood samples and ensuring correct storage are crucial for the accurate diagnosis and appropriate management of the condition.

## Data Availability

Not applicable.

## Abbreviations

PRIS: Propofol infusion syndrome
ASA: American society of anesthesiologists
Hb: Hemoglobin
pCO_2_: Partial pressure of carbon dioxide
ATP: Adenosine triphosphate
ADP: Adenosine diphosphate
PFK: Phosphofructokinase
L/P: Lactate/pyruvate
